# THE FUTURE OF MEDICINE, healthcare innovation through precision medicine: policy case study of Qatar

**DOI:** 10.1186/s40504-020-00107-1

**Published:** 2020-11-01

**Authors:** M. Walid Qoronfleh, Lotfi Chouchane, Borbala Mifsud, Maryam Al Emadi, Said Ismail

**Affiliations:** 1 Research & Policy Department, World Innovation Summit for Health (WISH), Qatar Foundation, P.O. Box 5825, Doha, Qatar; 2 Departments of Genetic Medicine and Microbiology and Immunology, Weill Cornell Medicine, Qatar, Doha, Qatar; 3 College of Health and Life Sciences, Hamad Bin Khalifa University (HBKU), Doha, Qatar; 4 Clinical Operations, Primary Health Corporation (PHCC), Doha, Qatar; 5 Qatar Genome Program, Qatar Foundation, Doha, Qatar

**Keywords:** Precision medicine, Population genomics, Cancer, Primary care, Healthcare research, Evidence-based research, Public health policy, National policy

## Abstract

In 2016, the World Innovation Summit for Health (WISH) published its Forum Report on precision medicine “*PRECISION MEDICINE - A GLOBAL ACTION PLAN FOR IMPACT*”. Healthcare is undergoing a transformation, and it is imperative to leverage new technologies to generate new data and support the advent of precision medicine (PM). Recent scientific breakthroughs and technological advancements have improved our disease knowledge and altered diagnosis and treatment approaches resulting in a more precise, predictive, preventative and personalized health care that is customized for the individual patient. Consequently, the big data revolution has provided an opportunity to apply artificial intelligence and machine learning algorithms to mine such a vast data set. Additionally, personalized medicine promises to revolutionize healthcare, with its key goal of providing the right treatment to the right patient at the right time and dose, and thus the potential of improving quality of life and helping to bring down healthcare costs.

This policy briefing will look in detail at the issues surrounding continued development, sustained investment, risk factors, testing and approval of innovations for better strategy and faster process. The paper will serve as a policy bridge that is required to enhance a conscious decision among the powers-that-be in Qatar in order to find a way to harmonize multiple strands of activity and responsibility in the health arena. The end goal will be for Qatar to enhance public awareness and engagement and to integrate effectively the incredible advances in research into healthcare systems, for the benefit of all patients.

The PM policy briefing provides concrete recommendations on moving forward with PM initiatives in Qatar and internationally. Equally important, integration of PM within a primary care setting, building a coalition of community champions through awareness and advocacy, finally, communicating PM value, patient engagement/empowerment and education/continued professional development programs of the healthcare workforce.

Key recommendations for implementation of precision medicine inside and outside Qatar:

1. Create Community Awareness and PM Education Programs
2. Engage and Empower Patients
3. Communicate PM Value
4. Develop appropriate Infrastructure and Information Management Systems
5. Integrate PM into standard Healthcare System and Ensure Access to Care

PM is no longer futuristic. It is here. Implementing PM in routine clinical care does require some investment and infrastructure development. Invariably, cost and lack of expertise are cited as barriers to PM implementation. Equally consequential, are the curriculum and professional development of medical care experts.

Policymakers need to lead and coordinate effort among stakeholders and consider cultural and faith perspectives to ensure success. It is essential that policymakers integrate PM approaches into national strategies to improve health and health care for all, and to drive towards the future of medicine precision health.

## Introduction

### Overview

#### PM in health care

A few years ago, Precision Medicine (PM) was just a promise that we all hoped to achieve. It is now a reality for some diseases like cancer, e.g., the use of the drug Imatinib (a.k.a. Gleevec), a small molecule kinase inhibitor, for chronic myeloid leukemia (CML). Here we define PM according to the most widely used definition from the President’s Council of Advisors on Science and Technology (PCAST): “*the tailoring of medical treatment to the individual characteristics of each patient to classify individuals into subpopulations that differ in their susceptibility to a particular disease or their response to a specific treatment. Preventative or therapeutic interventions can then be concentrated on those who will benefit, sparing expense and side-effects for those who will not*” (President’s Council of Advisors on Science and Technology (PCAST) 2008). This definition is consistent with the original one (Jain 2002), and adopted by The European Society for Medical Oncology (ESMO) Precision Medicine Glossary (Yates et al. 2018).

In a healthcare setting, the individual characteristics used for patient classification are of several types (Bilkey et al. 2019):

1. Traditional clinical phenotypes
2. Family history
3. Environmental and lifestyle contributors
4. Invariable patient-specific characteristics (genetic make-up)
5. Variable “omics” characteristics (cell type specific and vary over time)

The first two types of information are routinely recorded for each patient in a clinical setting. Environmental and lifestyle data are usually collected through questionnaires; however, these are often inaccurate. Increasingly, environmental and lifestyle measurements are available through wearable devices (Jeong et al. 2019), which are more practical and provide more detailed and often higher-quality data for PM purposes, though their integration with clinical data is still lacking. Data of categories from points 4 and 5 can be collected by high-throughput methods, such as next generation sequencing (NGS) techniques (Goodwin et al. 2016).

#### NGS technology

In 2003, the first human genome sequence was completed at a cost of USD $3.0 B. Since, NGS methods have gone through rapid technological development making cost a mere few thousand dollars. The continued global efforts to produce more accurate data, quicker and at an affordable price led to sequencing with clinically applicable turnaround time and radically reduced costs, e.g., genome sequencing can now be done for less than $1000. As a result, genomic data could be routinely integrated in health care. Other “*omics*” data are more difficult to obtain on routine basis due to higher cost, tissue specificity and variability over time. Reassuringly, many of these data are highly correlated with each other (Mitra et al. 2013), therefore current focus is on defining the most informative data and combination of features for routine tests. Additionally, disease-specific biomarker panels, derived from a variety of “*omics*” data, can be identified and used for diagnosis and potentially screening.

#### Population genomics

At a population level, scientific discoveries are often specific to the ethnic group studied with limited transferability, and most studies have focused on Western-European or North-American populations (Rao and Knowles 2019). Even global studies, such as the HapMap (Goldstein and Cavalleri 2005) or the 1000 Genomes Project (Birney and Soranzo 2015), that aim to understand human genetic variation, suffer from underrepresentation of certain ethnic groups. For example, until the start of the Qatar Genome project, there was no comprehensive information on the genomes of Arab populations. Today, despite a number of new initiatives, there is still a need to cover a large and ethnically rich region like the Middle East.

Isolated populations with high-level of endogamy are the most genetically distinct, and many of the early projects were in these nations, e.g., deCODE (Gudbjartsson et al. 2015) in Iceland, SardiNIA (Chiang et al. 2018) in Italy and later FarGen in Denmark’s Faroe Islands or in small nations like Estonia. However, recently there was a boom of population genomics projects amongst some of the larger nations as well. The UK leads the way with over 100,000 genomes already sequenced, but there are large-scale projects in the USA, China, Australia and Turkey among others.

The global promise of PM cannot be achieved if data being produced does not represent the human populations from various geographies and ethnicities. Developing countries do not have the means to take part in this international endeavor. One important example in this context is the H3Africa initiative “African genome project”, which is supported by US National Institutes of Health and the UK’s Wellcome Trust.

The rich data sets generated by these population genomics projects will need to be mined in an international effort to achieve the best health outcomes for all.

#### PM health economics

Health care budgets are limited, and decision-makers have to maximize the health outcome of the whole population with these resources. Thus, the implementation of PM in health systems largely depends on its perceived economic value. PM improves population health through diagnostic tests that stratify patients for more efficient or safer treatment. Drugs are prescribed only to the sub-population, which clinically benefit them, e.g., Gefitinib for patients with non-small cell lung cancer who carry activating *EGFR* mutations, and certain drug prescriptions are avoided for those who are likely to develop a serious adverse drug reaction (ADR), e.g., Azathioprine, an immunosuppressive medication, can cause severe neutropenia in those patients who have mutated *TPMT (*Gavan et al. 2018*)*. Prior to clinical practice introduction, a cost-effectiveness evaluation has to take place that compares the PM approach to other alternative strategies. In general, cost-effectiveness studies found that the value of diagnostic PM stems from its ability to achieve a more precise diagnosis, coupled with differential targeted therapy options based on it. Its value for population health also depends on the size of the sub-population affected by the test (Veenstra et al. n.d.).

#### PM challenges

There are a number of challenges facing PM implementation to harness its full potential. Below we capture some of the main challenges that present opportunities to improve.

##### Identification of biomarkers

Personalized interventions for the prevention of chronic diseases require robust evidence of efficacy and/or effectiveness of the new technology when implemented in health care. Validated biomarkers are key in this regard. The path to validated and qualified biomarkers is strewn with technical difficulties not to mention cost. The expectation is that some could be predictive to better target preventive interventions and avoid adverse events.

##### Economic evaluation of applications

In PM, diagnostic and informed medical decisions are based on an individual’s characteristics, including their clinical genomic profile. Cost-benefit analysis of genomic applications or tests remain crucial for wide deployment. Only those tests with proven efficacy and/or effectiveness and cost savings should be supported. Evidence of clinical utility provided by the tests is integral for acceptance, adoption and widespread usage. Moreover, ethical and social ramifications ought to be part of the clinical decision-making process, which could pose a challenge without policy framework or clarity.

##### Ethical, legal and policy issues

PM carries with it legal and ethical consequences. A dedicated effort is necessary to stimulate ethical, cultural and religious discussions, and implement responsible policies when dealing with health care dilemmas and testing results including facilitating cross collaboration among health care professionals. There is an increasing need also to develop, further harmonize and integrate dedicated policies into the existing health systems in a responsible manner, including offering education opportunities to families and professionals alike. Introducing a common ethically and legally validated policy framework could represent one of the drivers needed to manage a future with increasingly personalized healthcare and a shift in from disease treatment, to prevention to achieve a truly multidisciplinary approach that can realize the potential of PM.

### PM role in health

#### Rare diseases

Many of the 3500 or so genetic diseases are known to manifest symptoms within the first few months of a child’s life. Diagnosis normally is a slow process. The diagnostic odysseys result in suffering that might otherwise be avoided with a more rapid and comprehensive solution. NGS can provide information on virtually any gene in the genome. This cost effective test is capable of supporting the diagnosis of the majority of genetic diseases, thus, enabling clinicians to quickly determine the cause of rare diseases.

Genetic disorders represent a significant source of morbidity and mortality in populations with high rates of consanguinity, including in Arab populations. They are estimated to be the second leading cause of infant mortality in Qatar. Many of the genetic diseases that plague Arab populations are dissimilar to other populations; therefore, genetic testing developed for other populations are of very limited value for the Arab communities. This unmet need prompted stakeholders in Qatar to join their efforts to compile a list of conditions that are specifically documented to cause genetic disease in the Qatari population (Table 1) along with Qatari specific genetic variants revealed by NGS. This information resource provides a comprehensive genetic variant-disease reference database not only for Qatar but also for the Arab populations. In turn, this knowledge along with novel sequencing cases would be of increasing value for rapid diagnosis of new cases that present in the subsequent years. Table 1 shows few of these genetic disorders that have been integrated into the extended neonatal screening program in Qatar.

**Table 1:** Disorders integrated into the extended neonatal screening program in Qatar

Group Classification	Disorders
**Endocrinopathies**	Congenital hyperthyroidism Congenital adrenal hyperplasia Phenyl ketonuria (PKU) Benign hyperphenylalaninemia (HPA) Defects of biopterin cofactor biosynthesis (BS) Maple syrup disease (MSUD)
**Aminoacidopathies and urea cycle disorders**	Homocystinuria (HCY) Tyrosinanemia type 1 Citrulliniemia Argininosuccinic aciduria
**Organic acidurias**	Methylmalonic aciduria CBl-disorders Propionic aciduria Glutaric aciduria type I Isovaleric aciduria,3-methylcrotonulglycinuria Multiple acyl CoA dehydrogenase (MAD) deficiency Isobutryl-CoA dehydrogenase (IBDH) deficiency
**Fatty acid oxidation disorders,carnitine cycle defects anddisorders of ketogenesis**	Medium chain dehydrogenase (MCAD) deficiency Very long chain acyl CoA dehydrogenase (VLCAD) deficiency LCHAD deficiency Short chain acyl CoA dehydrogenase (SCAD) deficiency Carnithine transporter deficiency Carnithine palmitoyltransferase I (CPT I) deficiency Carnithine palmitoyltransferase (CPT II) deficiency 3-hydroxy-3-methyl-glutaryl-coenzyme A (HMG-CoA) lyase deficiency
**Others**	Ketothiolase deficiencies Classical galactosidase Biotinidase deficiency

#### Breast Cancer

Breast cancer, the most common cancer among women worldwide, is a highly complex, heterogeneous and multifactorial disease. A number of recognized risk factors contribute to breast cancer development. Above all, genetic factors play an instrumental role in augmenting risk (Table 2). Therefore, identification of breast cancer susceptibility genes has significantly improved care practice for cancer patients by targeting altered genes involved in carcinogenesis and treatment ineffectiveness.

**Table 2:** Breast Cancer- incidental BRCA1/2 breast cancer findings story

Prediction of genetic risk for disease is needed for preventive medicine. Decreasing NGS cost made genome-wide analyses affordable to assess variation in cancer susceptibility genes. The genome sequencing data provided by the Qatar Genome project and Qatar Biobank set the stage for cancer genomics studies in Qatar.	
Studying the genetic variation of cancer genes in the Qatari population, researchers from WCMQ, Sidra Medicine and HBKU-QCRI analyzed the genomes of 6000 Qatari. They obtained sequence details of more than 786 cancer genes including BRCA1 and BRCA2 that are known to be responsible of Hereditary Breast and Ovarian Cancers.	
The analyses revealed the presence of pathogenic BRCA1/2 mutations in 20 Qatari subjects. Given the high risk of breast and ovarian cancers in these individuals and/or other members of their families, a task force was formed by QPMI and HMC to setup a work plan for intervention and genetic counseling.	

It is well known that the characteristics of breast cancer differ among various patients. Indeed, substantial genomic changes often occur in disease progression from primary location to metastasis. Thus, it is the genomic analysis and characterization of several biomarkers that has revolutionized the clinical management of patients. These gene alterations represent novel target to engineer new therapies for breast cancer.

## Qatar initiatives

Personalized healthcare initiatives in Qatar are part of a coordinated and comprehensive strategy aiming to deliver world-class future health care. Two Qatar Foundation entities, namely Qatar Biobank (QBB) and the Qatar Genome Program (QGP) are at the heart of this strategy. Both have recently merged into one institute, the Qatar Precision Medicine Institute (QPMI), signaling a shift from supporting PM endeavors at the basic research level towards clinical implementation.

### Qatar biobank (QBB)

Established in 2011, QBB is the first population based biobank of the Gulf Region and one of the leading ones globally. The mission of QBB is to act as a central research platform in Qatar supporting biomedical research and pushing to translate outcomes into impactful preventive, diagnostic and therapeutic practices in Qatar. QBB aims to recruit more than 60,000 men and women Qatari nationals and long-term residents (> 15 years) aged ≥18 years. It collects multiple samples and comprehensive information on various health and lifestyle aspects (deep phenotypic and laboratory analysis data are collected from apparently healthy participants, including biofluids for future research purposes) and follows up its participants over a long period to record any subsequent health conditions.

Furthermore, QBB started recently to produce multi-omics data from thousands of its participant samples making the Qataris among the most studied populations at the molecular level. Such comprehensive data will provide scientists and clinicians with an unprecedented opportunity to have deep insights into the health of its population and thus have a strong basis to plan for the future of the health services in the country.

### Qatar genome project (QGP)

H.H. Sheikha Moza bint Nasser, Chairperson of Qatar Foundation, launched the vision for QGP at the WISH summit in December 2013. To date, QGP’s deliverables include; the sequencing of around 20,000 whole genomes mainly from QBB samples, assembling the QGP research consortium with around 150 local and international researchers to mine QGP and QBB data, and establishing genomic research funding schemes such as the path towards precision medicine (PPM) awards in collaboration with the Qatar National Research Fund (QNRF). Other initiatives include conducting benchmarking surveys for the general public and health care professionals to gauge public awareness and attitudes towards genomic medicine and to identify gaps in the health care system to bridge before comprehensive implementation. In addition to, initiating local graduate programs in genetic counseling, genomic medicine and various other educational and awareness schemes. (see Future Directions section on implementation and education).

**Fig. 1:**
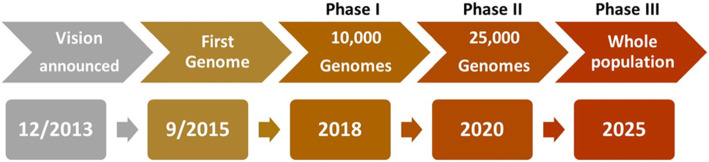
Timeline for Qatar Genome Project (QGP)

The current phase of the project, Phase II, will witness the sequencing of almost 10% of the Qatari population. With the comprehensive phenotypic data collected at QBB, this would provide an amount of health related data that few populations around the world would have. In 2021, Phase III of the project, the population phase, will commence aiming at sequencing of 100,000 whole genomes (Fig. 1). In this phase, samples will be also collected from primary, secondary and tertiary health care centers in Qatar.

### Evolution to Qatar precision medicine institute (QPMI)

QGP and QBB have made great strides towards preparing the scene for the next level of PM implementation at a national level. This paved the way for the creation of a new entity that would consolidate national efforts and resources to harness all achievements and steer in a coordinated manner towards the realization of the PM strategy for Qatar. This new QF entity is the Qatar Precision Medicine Institute (QPMI).

QPMI will be Qatar’s focal point where all national stakeholders can come together to help move Qatar into being a global leader in the clinical implementation of PM. It will capitalize on the great wealth of data generated by QBB and QGP and the research results that are giving unprecedented key insights to translate into impactful clinical measures directly affecting the health of the local population.

QPMI is working closely with local stakeholders and health care providers on specific first model examples of clinical implementation of PM. One of these examples is the Q-Chip (Table 3). It contains variants extracted from the whole genome data provided by Qatar Genome. The second, more refined version of the Q-Chip V2 will be used to analyze clinical samples by Hamad Medical Corporation (HMC). Early examples, include delivering genomic reports to thousands of QBB participants on dozens of traits related to general health and wellness besides selected pharmacogenomics variants associated with the metabolism commonly used drugs prescribed for cardiovascular disorders, pain management, and cancer.

**Table 3:** Qatar Precision Medicine - from basic research to clinical implementation - the Q-chip

Qatar Genome led national efforts to produce the first version of a Qatar microarray gene chip in 2018. This chip included hundreds of thousands of gene variants and disease associated mutations to be used both for research and in clinical diagnosis. The chip content was based on Qatari genetic and genomic data. The Q-Chip was the result of a collaborative work led by Qatar Genome and involving major local stakeholders including QBB, WCMQ, HMC and Sidra Medicine.	
The Q-chip is an example of how data provided by a large-scale genome project like QGP can start to deliver impact at the clinical care level and lead efforts to introduce precision medicine practices into the health care system. The Q-chip will provide more accurate genetic testing for a wide range of disorders based on data that is more relevant to the local population. Currently, Qatar Genome is working with other stakeholders and the chip manufacturer (Affymetrix/Thermofisher) to produce the second version of the Q-Chip. This version will have more refined clinical content and design to suit the local needs delivering on the promise of precision medicine for the population and precision healthcare in Qatar. A prototype, the first ‘Q-chip’, was presented to Her Highness Sheikha Moza during the WISH 2018 Summit.	

## Future directions

### Implementing Precision Medicine

Current medicine mainly uses a one-size-fits-all approach. PM transforms the way medicine is practiced and delivered. PM has the conviction that the same disease manifestation can have a different cause, course or therapeutic efficacy, depending on the patient. Therefore, an individualized approach to each patient is crucial. PM utilizes clinical, genomic, environmental and lifestyle data that are unique for each patient to prevent, diagnose- including early detection- or decide on disease treatment (Twilt 2016; Carter and He 2016).

In order to integrate PM into healthcare practice (Pritchard et al. 2017), the following principles should be considered:

➢ Healthcare providers, payers, employers and policymakers, as well as patients and their families, need to have a better understanding of PM concepts and technologies.
➢ Policies and practices related to patient engagement, privacy, data protections and other ethical, legal, and societal issues regarding the use of individual molecular information must be addressed.
➢ Best practices must be established for the collection and dissemination of evidence demonstrating clinical utility of PM and ensure the recognition of its value to care.
➢ Effective healthcare delivery infrastructure and data management systems should be developed and applied so that individual patient and clinical support information is comprehensive, useful and user friendly in order to guide clinical decisions.
➢ Best practices for healthcare delivery approaches, processes and program operations that ensure access to PM must be established and implemented.

Currently, a limited number of studies or surveys have been conducted that addresses infrastructure requirements and challenges for implementing PM. Recently, the Qatar Genome conducted two national surveys to help better plan for future PM implementation in Qatar. The first was a population wide survey to gauge public awareness and attitudes, while the other one included health care professionals to identify gaps in the health system that need to be abridged. The main outcomes of those surveys (Abdul Rahim, H F et al. *J. Hum. Genet.*, 2020, 10.1038/s10038-020-0806-y) is summarized below:

1. Overwhelming support and positive attitude towards the genome project regardless of gender, age and education in the population sample (range: 58–71%). Further, up to 83% of those surveyed expressed desire to test for specific non-communicable diseases risk.
2. The vast majority of participants (81%) expressed interest to learn more about their genetic makeup primarily for health reasons; moreover, a significant proportion of them (> 70%) showed willingness to donate samples to Qatar Genome national research initiative.
3. Over 90% of health care professionals surveyed believed that adopting PM practices will provide better service to their patients. However, they identified major obstacles that they might face along the way. The two main challenges were the need for more training and lack of time to enroll in such programs. Also, they articulated the need for more genetic counselors to be incorporated within the health system to act at the interface with patients.

However, globally a number of potential challenges and barriers surfaced for deliberation locally and internationally. These include:

1. Lack of awareness, knowledge and value recognition within the healthcare organizations and the public at large regarding PM practices, policies and community support or information is not readily available.
2. Clinical and economic data demonstrating value of new technologies are not clear.
3. Racial, ethnic, economic and regional disparities are not appropriately addressed.
4. Providers do not adequately involve patients/families in their healthcare decision-making, patient preferences and prevention strategies.
5. Communication across the continuum of care is insufficient or breaks down easily.
6. Medical school curricula on PM are often outdated. Clinical practice guidelines are based on traditional medicine and often do not include molecular and genetic data.
7. Preventive/primary care is frequently overlooked in practice.
8. Important infrastructures for PM such as genomics, high-value diagnostic tests and services, electronic health records are not well established.

### Building the Foundation for Precision Medicine in the State of Qatar

Genetics is an important contributor to the complexity of human physiology. Distinct genetic variants cause conditions that respond to different treatments yet share a similar set of symptoms. Without a molecular to determination, it might not be possible to determine which treatment will be most effective (Aronson and Rehm 2015; Freimuth et al. 2017). Understanding the patient’s genomic make-up is crucial for providing optimal care for many diseases. Clinicians now have access to an increasing array of tests at their disposal that allow them to assess the condition of their patients. QBB is the custodian of samples and information on health and lifestyle from the Qatari population. The Qatar Genome initiative is mapping the genome of the local population. These enable the identification of genotype-phenotype associations relevant to the Qatari population. To reiterate, the wealth of such data accelerates innovation and empowers researchers to make breakthrough discoveries as well as help policy makers to better plan for future health care directions in Qatar. This will provide unique insights that would enable the development of precision healthcare in Qatar to usher in a new era in patients’ centric care.

### Integrating precision medicine technology into primary care

With their longitudinal patient relationships, primary care physicians and their care teams are uniquely situated to promote preventive medicine, including cancer screening, management of common chronic diseases such as diabetes and hypertension, and health education. PM is beginning to gain ground within the healthcare industry though not all patients are benefiting from these advances (Ersek et al. 2018) or healthcare officials have been wrestling with the challenge of leveraging insights from genomics (Cooper-DeHoff and Johnson 2016). Yet, PM remains undeniably the field’s future (Table 4). Incorporation of medication choices and PM technology into primary care has significant potential to lead to improved clinical outcomes. Examples of implementing PM technologies into primary care include cancer screening, premarital testing and genomic sequencing.

**Table 4:** Diagnosis of Diabetic Neuropathy- artificial intelligence (AI) meets precision medicine

Precision medicine aims to deploy biomarkers to achieve rapid diagnostic and prognostic capability to enable targeted and more efficacious treatment.	
Diabetes affects ~ 20% of the Qatari population causing diabetic neuropathy (DN) in ~ 35% of the cases and remains undiagnosed in ~ 80% of these patients. Researches from WCMQ, QU and HMC have pioneered corneal confocal microscopy (CCM) diagnostic, a non-invasive, ophthalmic imaging technique to identify early subclinical nerve degeneration and regeneration after therapeutic intervention. They used machine learning technology to achieve rapid automated quantification of corneal nerve fibers to clinically classify patients with high sensitivity and specificity.	
This work translates the AI outcome into a clinically meaningful outcome for rapid objective diagnosis of early diabetic neuropathy enabling risk factor reduction to prevent progression of DN to foot ulceration and amputation. It also has a wide range of other diagnostic and prognostic applications in neurology as CCM can identify neurodegeneration in several neurodegenerative conditions.	

PM complements primary care and family physicians work. The debate concerning whether PM ought to be integrated into primary care is ongoing (Fodor and Karnieli 2016; Ginsburg and Phillips 2018). In cases where a patient can acquire their genomic profile online data minus guidance from doctors might be dangerous. The primary care setting in the future may add a new specialty, epigenetic experts who are a combination of computer programmers and physician assistants (Hassen and Khomani 2019).

The nature of PM may present numerous barriers to the incorporation in primary care. The first hurdle involves the vast complexity and the need for physicians to learn about a significant range of new tests and novel ways to counsel patients through probability-based decision. Another barrier, medical education, professional development and instruction in PM technology to primary care physicians are vital to PM penetration and adoption.

#### Precision medicine in Cancer screening in primary health care

A confluence of forces is driving the demand for the personalization of cancer screening recommendations. Recommendations are increasingly based on individual patient preferences, medical history, genetic and environmental risk factors, and level of interaction with the healthcare system. Current examples include choices between colonoscopy, fecal testing, and emerging tests for colorectal cancer screening; the use of genetic information and availability of home self-testing in cervical cancer screening; the integration of multiple risk factors and patient preferences to decide the intensity and length of breast cancer screening; and the issues of smoking cessation and finally, competing priorities when deciding whether or not to pursue lung cancer screening (Pernas et al. 2017; Selby et al. 2018).

**Fig. 2:**
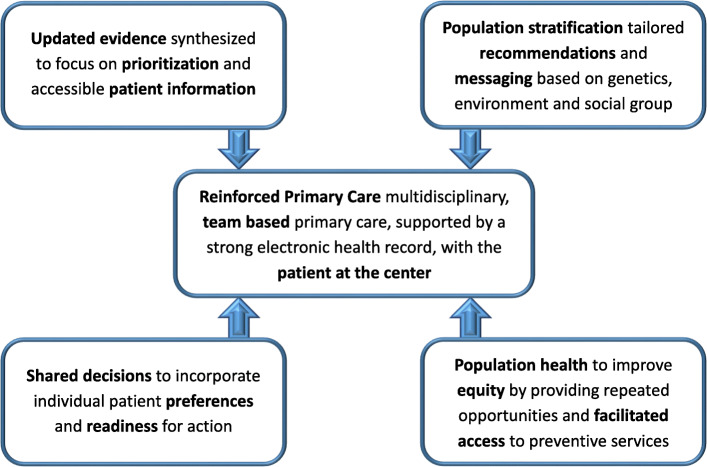
Primary Care and Personalized Cancer Screening

Examples of how primary care can be augmented to make personalized cancer screening a reality is illustrated in Fig. 2. On the other hand, Table 5 depicts screening program progress.

**Table 5:**
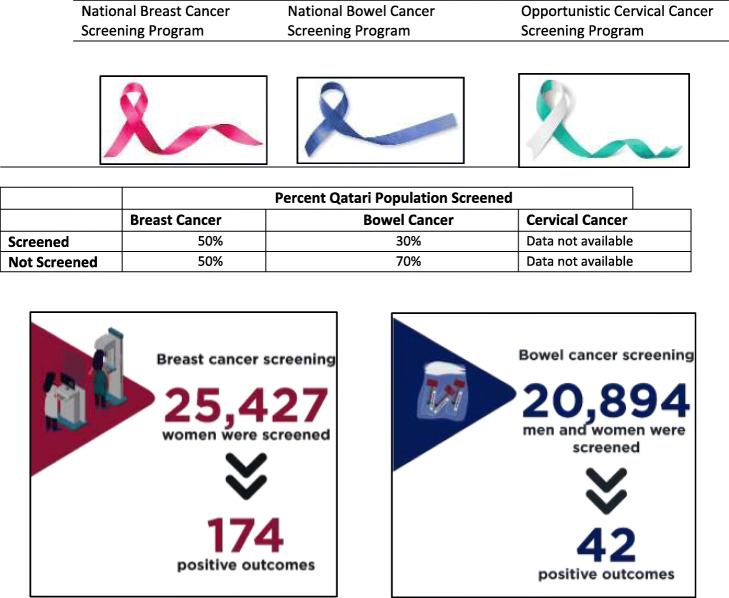
Primary Health Corporation (PHCC) national cancer screening programs and outcomes

### Gaps, challenges, and opportunities

The overview section identified certain gaps and presented few of the challenges encountering PM to become mainstream in medical practice and public health. Personalized interventions for the prevention or treatment of diseases require robust evidence of efficacy and/or effectiveness of the new technology when implemented in a health care setting.

#### Biomarkers for specific therapies and for chronic diseases prevention

PM uses an individual’s genomic information as part of their clinical care. Specific biomarkers are used to stratify populations to assess an individual’s risk or resistance to disease as well as their potential response to different treatments. This classification is reliant on companion diagnostics. Nowadays, there is a tendency towards moving away from the “*one test, one drug*” model that has defined companion diagnostics to utilize biomarker panels. There is also an expectation that this may lead to better targeting of preventive interventions by defining the disease and targeting the treatment based on a person’s molecular pathology (Boccia et al. 2019).

#### Optimizing precision medicine applications

The penetration of genomic technologies into the clinics is contributing to the shift towards PM, where medical decisions are based on an individual’s characteristics, including their genomic profile. This has made their performance evaluation crucial to deploy in practice. Genomic applications with evidence of efficacy, effectiveness and cost-effectiveness should be utilized in clinical and public health practice (Aronson and Rehm 2015). A comprehensive evaluation of the value outcomes of the new technology should also include evidence on the social aspects, and context-related dimensions to better support the clinical decision-making process (Kessler 2019).

#### Ethico-legal and policy issues

The implementation of genomic testing in PM carries legal and ethical consequences. Clinical implementation has dealt an ethical dilemma to practitioners both in the West and in the Islamic World. The two main ethical predicaments revolve around the extent of information sharing: (1) significance and interpretation of findings called “variants of unknown significance” and (2) results disclosure of unintended findings termed “incidental findings”. Religion plays a key role in this region at all levels. There is a necessity to consider: the family need for genetic counseling, the marital consequences and the effects on offspring. To benefit from PM, stakeholders in genetic testing and family members’ notification need to be carefully aligned (De Paor and Blanck 2016; Korngiebel et al. 2017).

#### Sociotechnical analysis concerns

Indeed, personal genomics analysis is a conundrum. There are ethical complications of testing that are not clear at all levels. It presents a dilemma in terms of What to report? How to report it and When to report it? Undoubtedly, there are still issues that need to be contended with such as personal privacy and data management security, not to mention the educational component of conveying information as the subject needs to be well informed and learn how to deal with the results. Moreover, from a scientific standpoint the predictive value for the vast majority of common health conditions is weak. We are still a distance from resolving these matters at the individual, societal, and regulatory levels (De Paor and Blanck 2016; Korngiebel et al. 2017).

#### Organizational models

The integration of genomics in other medical specialties should be promoted through new delivery models involving different healthcare professionals (medical specialists, nurses, technicians, etc.) and new professional roles (i.e., genetic counselors and genetic nurses), to guarantee the use and sustainability of existing and new genomic applications in practice. Additionally, roles and responsibilities need to be redistributed among different health professionals to enhance work performance and standards of care. It is advisable to define the appropriate model for genetic service provision in a specific setting according to the type of healthcare system and the genetic test provided (Stouffer and Lee 2019; Povsic et al. 2019). Professional education/training in genomics medicine, laboratory quality standards, and public awareness are essential factors for the successful implementation of genomic applications in practice.

### Investing in building capacity

Advancement of PM implies ensuring systems of healthcare adopt the most technologically and scientifically appropriate techniques in disease management (Kłak et al. 2016; Martinez-Garcia et al. 2019; Levit et al. 2019). Capacity building for the adoption of PM could be achieved through various institutions such as universities, which improve the skills, tools, knowledge, and equipment vendors to implement PM. Investing in building capacity for PM implementation is vital (Hellings et al. 2019). The universities may enhance capacity building through teaching various PM courses/workshops such as genomics, bioinformatics, genetic counseling etc.

#### Bioinformatics

Bioinformatics remains fundamental to PM to make a final diagnosis (Kim et al. 2019). Clearly, an efficient bioinformatics architecture is required in supporting PM to analyze, manage, as well as record all the information collected (Levit et al. 2019). It must be emphasized that PM’s success relies on the access of quality patient data (Kessler 2019).

#### Genomics Counselling

Genomic counselors possess specialized education. Their knowledge offers personalized information that assists people to make informed decisions concerning health. Counseling supports implementation of PM and may have a significant impact on public health practice at large as the population shares their health information (Mahesh 2019).

#### Clinical genetics

Clinical genetics involves the diagnosis and management of inherited illnesses. Genetics and bioinformatics combine to confirm accurate diagnostics, and ensure proper, early treatment, thus, evading unnecessary cost and wasted time.

#### Raising awareness of precision medicine

Raising awareness about PM and how it can be used to deliver better health care is extremely important in efforts to further integrate PM into clinical practice.

#### Public awareness and engagement campaigns

Efforts are needed to raise understanding of PM, through public awareness and engagement campaigns. Warner et al. (Warner et al. 2018), argue that the greater availability of information for the public, the higher the chance of creating awareness amongst them considering genomics technical complexity and PM approaches. The public ought to be made aware of the customization of healthcare as misinformation and propagation of fake news through social networks, may distort PM value (Whitcomb 2019).

#### Trained community volunteers

In some cases, raising awareness is done through trained community volunteers “influencers” who are qualified to offer impactful connections to the healthcare systems and the community as they deliver and disseminate health information to the public since the method enhances public knowledge (Roda et al. 2018). The volunteers engage communities through public communication/education campaigns to explain PM concept (Kessler 2019). These individuals address the continuing gaps in health literacy, thus empowering community members to make informed decisions regarding PM management, therapies and research.

#### Project public acceptance

This concept involves building consensus within the society concerning enhanced awareness of PM. The project relies on the Ministry of Education curriculum being taught at schools or higher institutions. The inclusion of PM concepts in the school curriculum will help people learn about it from a tender age and getting to understand the customization of healthcare (Povsic et al. 2019). Many governmental initiatives to gain public acceptance may be achieved through school visits, where the students are informed on the pros and cons of PM as well as its application to a patient (Sciurba et al. 2016).

### Continuous education of health providers and professionals

Engaging healthcare professionals is important for the transition towards a PM-based health care. Most healthcare practitioners have not had a formal training in genomics and other omics-based technologies that provide the means for precision medicine. Therefore, they do not feel comfortable with ordering such tests or interpreting the results for their patients. Upon launching the QGP project, Qatar has invested in graduate-level training of professionals who will be able to make use of the genomic data (Table 6).

**Table 6:** Precision medicine education story – a nurse perspective

I work as a Staff Nurse in the cardiac rehabilitation unit at the Heart Hospital, HMC. I completed my undergraduate study in nursing at the University of Calgary in Qatar, and recently completed my MSc degree in Genomics and Precision Medicine at HBKU.	
My goal at the cardiac rehabilitation is to promote and educate patients about heart disease risk factors and about healthy life style. This program helped me to build my knowledge, and gave me the tools I needed to be more confident at performing my job at HMC. By incorporating what I learned at HBKU in patients’ care plans, we can provide tailored, personalized care for each patient.	
My plans involve doing more research related to heart disease using precision medicine.	
PhD Candidate Genomics and Precision Medicine, HBKU	

#### HBKU program

The Genomics and Precision Medicine (GPM) MSc and PhD programs at the Hamad Bin Khalifa University (HBKU) aim to develop researchers who can derive new genomics insights that can be translated into the clinics. Table 7 displays the GPM program enrollment and graduation since inception to illustrate capacity development (a total of 18 to date). The graduates (11 in total) have been placed locally throughout Qatar healthcare system mainly Sidra Medicine, Hamad Medical Corporation and Ministry of Public Health, whereas 7 graduates made the decision to pursue their PhD in the GPM program. See Table 6 as an example of a nurse working at the Heart Hospital, HMC expressing her program experience and aspirations on the job.

**Table 7:** The Genomics and Precision Medicine (GPM) Programs at the Hamad Bin Khalifa University (HBKU)

	2017–2018	2018–2019	2019–2020	2020–2021
**Enrolled**				
MSc	10	10	11	10
PhD	12	12	12	11
**Graduated**^a^				
MSc	–	10	8	–
PhD	–	–	–	–

^a^Note: graduated students placed locally throughout Qatar healthcare system or enrolled in the GPM PhD program, see Table 6 as an example

#### QU program

Qatar University (QU) offers MSc in Genetic Counselling that trains professionals who can effectively advise patients and their families about a range of genetic conditions.

While these programs help with capacity building for PM, they do not fully address the knowledge gap for healthcare practitioners, as there are only few of them, who can take a sufficiently long time out to enroll in these programs. Shorter, more targeted training opportunities are required to reach a broader medical audience. Continuing Professional Development (CPD) activities and multi-module courses are ideal for that. HBKU has recently held its first CPD symposium on “*Genomics in Clinical Applications*” to start a series of genomics and PM related CPD activities.

## Key policy recommendations

We make the following key policy recommendations that are applicable inside and outside of Qatar. Moreover, we share the successful personalized cancer screening framework in primary care learned from the Qatar experience. Key Recommendations for Implementation of Precision Medicine in The State of Qatar and other countries are the following:

### Value recognition

➢ Conduct PM economic impact studies that are meaningful to stakeholders.
➢ Develop standards as well as measurable targets for comparative effectiveness studies.
➢ Design and implement research studies that demonstrate the cost and benefits in areas of unmet need.
➢ Develop and implement proactive policies that incentivize healthcare providers for optimizing treatments based on individual patient characteristics.

### Infrastructure and information management

➢ Coordinate institutional policies and processes that assure effective communications through the continuum of care and across research and clinical programs and develop an effective process for making programmatic decisions.
➢ Develop proactive policies to incentivize data sharing and facilitate real-time data exchange for learning health systems.
➢ Include each patient’s clinically actionable variants within electronic health records.
➢ Assure that all medical data, clinical support and outcomes information are captured, standardized and interoperable across multiple platforms.
➢ Develop and implement customizable, user-friendly platforms to input data and provide clinical support information to physicians in a way that saves time and resources.

### Ensuring access to care

➢ Develop policies that ensure clinical guidelines and support technologies are focused on providing the best treatment strategies for individual patients and are regularly updated.
➢ Ensure that professional fees for PM services are adequate.
➢ Ensure access to experts and counselors where appropriate streamlining the process for their inclusion in patient care.
➢ Develop and implement a PM healthcare system-wide approach and delivery models.

### Creating community awareness and education programs

➢ Develop online educational information and social media platforms to raise awareness of PM events, activities and new technologies that address stakeholders’ needs.
➢ Organize collaborative forums to develop and agree upon a common lexicon regarding PM and agree upon consistent themes for communications based on scientific evidence and value.
➢ Provide healthcare professional groups and patient support organizations with PM information and educational materials, and actively manage multiple communication and dissemination channels.
➢ Identify physician, pharmacists and community leaders to engage patients and participate in regional events that raise awareness and promote precision medicine.
➢ Update medical and pharmacy schools’ curricula to integrate PM concepts and develop PM Continuing Medical Education (CME) programs.

### Patient engagement and empowerment

➢ Include patient representatives in the development of proactive policies and practices related to patient protections and the use of individual molecular information.
➢ Implement state-of-the-art cyber-security measures related to individual molecular information.
➢ Develop programs to explain diagnostic test results to patients; provide them with recommendations and easy access to related information and counseling services.
➢ Incorporate patient-reported outcomes through multiple channels to capture and better understand patient experiences.
➢ Design clinical trials with diverse research participants that include persons of various ethnicities, races, ages and genders to better inform the value of a specific treatment option for any given patient.

## Conclusion

Preparing the public, healthcare professionals and policy decision-makers to the future of medicine is critical. Incrementally, the healthcare system is departing from traditional medicine. The promise of PM is so great, for a moment it seems so intimidating. Distinctively, it builds on data, analytics and AI. It also requires trust amongst stakeholders and patients to harness the benefits of precision medicine.

The value and success of PM is evident in cancer treatment and genomics, and PM will continue to do so. However, organizations have shown limited establishment of PM programs in the past, with implementation cost and lack of expertise as the most commonly cited barriers. Additional barriers include limited resources, test turnaround time, lack of knowledge amongst healthcare professionals and communication challenges. In summary, despite the potential of precision medicine to transform healthcare, the adoption of PM in practice has been slow, in large part due to the lack of evidence of clinical utility provided by tests. Other interrelated factors include evidence of test cost-effectiveness, limited insurance coverage, and inadequate regulatory framework.

It is essential that policymakers integrate PM approaches into national strategies to improve healthcare. These approaches will ultimately merge with efforts to address rising rates of non-communicable diseases (NCDs), expand health data infrastructure and connectivity, develop genomic data policy, increase diagnostic capacity, and create biobanks that hold both physical and digital resources.

Technology and innovations in medicine aim to help all patients globally, providing evidence for particular conditions that need to be personally considered, involving the patient’s decision while treating, predicting and preventing disease. The purpose should be to have precision medicine available everywhere at any time for everyone.

## Data Availability

All data generated or analyzed during this study are included in this published article.

## Abbreviations

ADR: Adverse Drug Reaction
BRCA: BReast CAncer gene
CCM: Corneal Confocal Microscopy
CME: Continuing Medical Education
CML: Chronic Myeloid Leukemia
CPD: Continuing Professional Development
DN: Diabetic Neuropathy
EGFR: Epidermal Growth Factor Receptor
GPM: Genomics and Precision Medicine graduate program
HBKU: Hamad Bin Khalifa University
HMC: Hamad Medical Corporation
NGS: Next Generation Sequencing
PHCC: Primary Health Corporation
PM: Precision Medicine
PPM: Path towards Precision Medicine awards
QBB: Qatar Biobank
QCRI: Qatar Computing Research Institute
QGP: Qatar Genome Program
QNRF: Qatar National Research Fund
QPMI: Qatar Precision Medicine Institute
QU: Qatar University
TPMT: Thiopurine S-methyltransferase
WCMQ: Weill Cornell Medicine-Qatar
WISH: World Innovation Summit for Health
